# Changing trends in elephant camp management in northern Thailand and implications for welfare

**DOI:** 10.7717/peerj.5996

**Published:** 2018-11-23

**Authors:** Pakkanut Bansiddhi, Janine L. Brown, Chatchote Thitaram, Veerasak Punyapornwithaya, Chaleamchat Somgird, Katie L. Edwards, Korakot Nganvongpanit

**Affiliations:** 1 Center of Excellence in Elephant and Wildlife Research, Faculty of Veterinary Medicine, Chiang Mai University, Chiang Mai, Thailand; 2 Center for Species Survival, Smithsonian Conservation Biology Institute, Front Royal, VA, USA; 3 Department of Companion Animal and Wildlife Clinic, Faculty of Veterinary Medicine, Chiang Mai University, Chiang Mai, Thailand; 4 Department of Food Animal Clinic, Faculty of Veterinary Medicine, Chiang Mai University, Chiang Mai, Thailand; 5 Excellent Center of Veterinary Public Health, Chiang Mai University, Chiang Mai, Thailand; 6 Center of Excellence in Veterinary Biosciences, Department of Veterinary Biosciences and Public Health, Faculty of Veterinary Medicine, Chiang Mai University, Chiang Mai, Thailand

**Keywords:** Asian elephant, Management, Elephant camp, Welfare, Tourism, Thailand

## Abstract

**Background:**

Elephant camps are among the most attractive destinations in Thailand for tourists from many countries. A wide range of management strategies are used by these camps, which can have varied impacts on health and welfare of elephants.

**Methods:**

This study surveyed 33 camps with 627 elephants in northern Thailand to quantify the types of management practices and work activities experienced by captive elephants. The survey consisted of an interview with camp owners, and direct observations of camp operations.

**Results:**

Data revealed considerable variation in elephant demographics, work activities, elephant care (i.e., housing, restraint, nutrition, health care, and breeding), and mahout management among the camps. In general, older camps (those in existence for >16 years) were involved in more intensive activities, like riding with saddles and shows. By contrast, newer camps provided more one-on-one activities for tourists and elephants, and emphasized more intimate, relaxing experiences (e.g., feeding, bathing, walking) than entertainment. A demographic shift also was observed, with elephants 20 years of age and younger having a sex ratio closer to 1:1 compared to elephants in older age categories (1:4.1–1:9.8).

**Discussion:**

Shifts in elephant management to less intensive activities were observed, which could have positive implications for elephant welfare. The shifting sex ratio suggests successful captive breeding is resulting in the birth of more males, which could present new welfare challenges in the future, because bulls can be more difficult to manage and socialize, and are more likely to be kept isolated during musth. Ultimately, the goal is to understand how camp activities affect welfare, and to develop science-based guidelines and standards to aid in the management of both male and female elephants used in tourism.

## Introduction

The Asian elephant is categorized as endangered by the International Union for Conservation of Nature and is listed under Appendix I by the Convention on International Trade in Endangered Species of Wild Fauna and Flora. For centuries, elephants throughout Asia have been the pride of kings and used as objects of worship, beasts of burden, machines of war, and working animals in the logging industry (Lair, 1997; Schliesinger, 2015). The 13 range states of Asian elephants hold a current population estimated at 45,000 elephants, with approximately 15,000 elephants in captivity (Sakamoto, 2017). In Thailand, the elephant is a national symbol and an integral part of Thai and Buddhist culture. In the early 1900s, it was estimated there were 100,000 captive elephants in Thailand, almost all of them involved in the logging industry. However, as logging destroyed natural habitats and the need for captive elephants diminished, numbers plummeted to only 3,400 by 1985 (Schliesinger, 2015; Tipprasert, 2001). Continued deforestation led to flash floods in the southern provinces in late November 1988, after which the Thai government imposed a nationwide logging ban through an emergency decree in January 1989. This resulted in thousands of elephants suddenly becoming unemployed (Collins, 1991; Laohachaiboon, 2010). Now out of work, many mahouts (i.e., elephant handlers) took their elephants to large cities, like Bangkok, to beg for food and entertain tourists, resulting in poor welfare conditions, including problems of inadequate nutrition, unnatural living conditions, and elephants being hit by cars (Pimmanrojnagool & Wanghongsa, 2001). To rectify this situation, a law banning elephants from the streets of Bangkok was passed in 2010, and commercialized elephant camps mushroomed in tourism destinations throughout the country (Schliesinger, 2015).

Recent estimates of elephant numbers in Thailand indicate there are about 3,500 wild elephants in 69 protected areas, and a similar number in captivity, mostly (95%) privately owned (Asian Elephant Specialist Group, 2017). The majority of captive elephants in Thailand are in the north and northeast of the country (∼60%), primarily in Chiang Mai province (Godfrey & Kongmuang, 2009).

Elephant tourist activities often involve interacting with the public in the form of shows, trekking, bathing, feeding, painting, and other activities, and are not closely monitored or regulated. The law pertaining to “domestic” elephants is the Beast of Burden Act of 1939, in which they are classified as draft animals along with buffalo, oxen, donkeys, and other livestock. The Act allows elephants to be treated as private property, and infers no animal welfare protection. Thailand has a registration system managed by the Ministry of Interior (Department of Provincial Administration), but it is not required until the elephant reaches 8 years of age. The Ministry of Agriculture and Cooperatives (Department of Livestock Development, DLD) is responsible for elephant movements and health care through livestock veterinary networks, and coordinates a microchipping program. Currently, there are two welfare laws that have the ability to protect captive elephants in the tourist industry: the Criminal Code B.E.2499 (A.D.1956) (section 381 and 382) and the Prevention of Cruelty and Animal Welfare Provision Act B.E.2557 (A.D.2014). These laws protect all domestic animals in general, not captive elephants specifically. Moreover, they are vaguely defined, and the maximum fines (1,000 baht for the Criminal Code B.E.2499 and 40,000 baht for the Act B.E.2557) have done little to ensure compliance. There also are two elephant camp standards, one issued by the Department of Tourism under the Ministry of Tourism and Sports, and the other by the DLD that include regulations on elephant shelters, health care, food and water, mahout management, environmental and waste management, tourist service and safety, and recording systems. However, many aspects related to welfare are not covered, nor are they based on scientific evidence, and again, there is a lack of proper enforcement. More recently, a 20-year National Elephant Conservation Action Plan (2018–2027) was drafted, which includes language related to establishing health care centers in four regions of Thailand, and conducting training courses on elephant health and welfare for camp owners, mahouts and vet assistants. This action plan will provide guiding principles for Thai elephant conservation over the next two decades.

The few surveys that have been conducted on elephant camp management found elephants to be exposed to a variety of demographic, work, husbandry, and living situations, some of which may affect health and welfare (Chatkupt, Sollod & Sarobol, 1999; Godfrey & Kongmuang, 2009; Kontogeorgopoulos, 2009a, 2009b; Schmidt-Burbach, Ronfot & Srisangiam, 2015). Chatkupt, Sollod & Sarobol (1999) surveyed 87 elephants in three regions (Bangkok, Phuket, Chiang Mai) and summarized camp type (seasonal, permanent), work (show, taxi, trekking, street wandering), and adequacy of shade, food, sanitation, and footing, and how those correlated to biological indicators (general behavior, body condition, and ear, tail and trunk activity). Except for the lower body condition of elephants in Bangkok, health indicators were not affected by work or rest conditions, but were positively related to the amount of shade. In general, elephants working in Chiang Mai were in better body condition, and had adequate shade, footing conditions and better work hours. Kontogeorgopoulos (2009a, 2009b) indicated welfare problems in six areas: lack of an appropriate set of laws, suffering injuries related to working, poor nutrition, limited social environments, improper training, and the declining quality of mahouts.

In recent years, the welfare of captive elephants has become a topic of intense debate among tourists, scientists, animal welfare groups and stakeholders on a global level. Schmidt-Burbach, Ronfot & Srisangiam (2015) evaluated 106 elephant venues in Thailand and concluded most animals were kept in inadequate conditions, based on low scores for freedom to move (i.e., use of chains, although length of time was not defined), access to veterinary care, environmental quality, hygiene standards, and work intensity. One can debate if subjective claims that entertainment activities (shows, riding), presence of hooks (ankus), or use of chain restraints are always negative, especially when they are not based on findings related to specific welfare outcomes, but it is clear that strategies used to manage elephants in tourism are diverse and may not always meet animal welfare needs. Thus, this study aimed to identify the current range of activities and management practices experienced by captive elephants, focusing on tourist camps in northern Thailand. Special attention was paid to differences related to camp size, types of work, and years in operation to determine trends in elephant tourism.

## Materials and Methods

### Data collection

**Figure 1:**
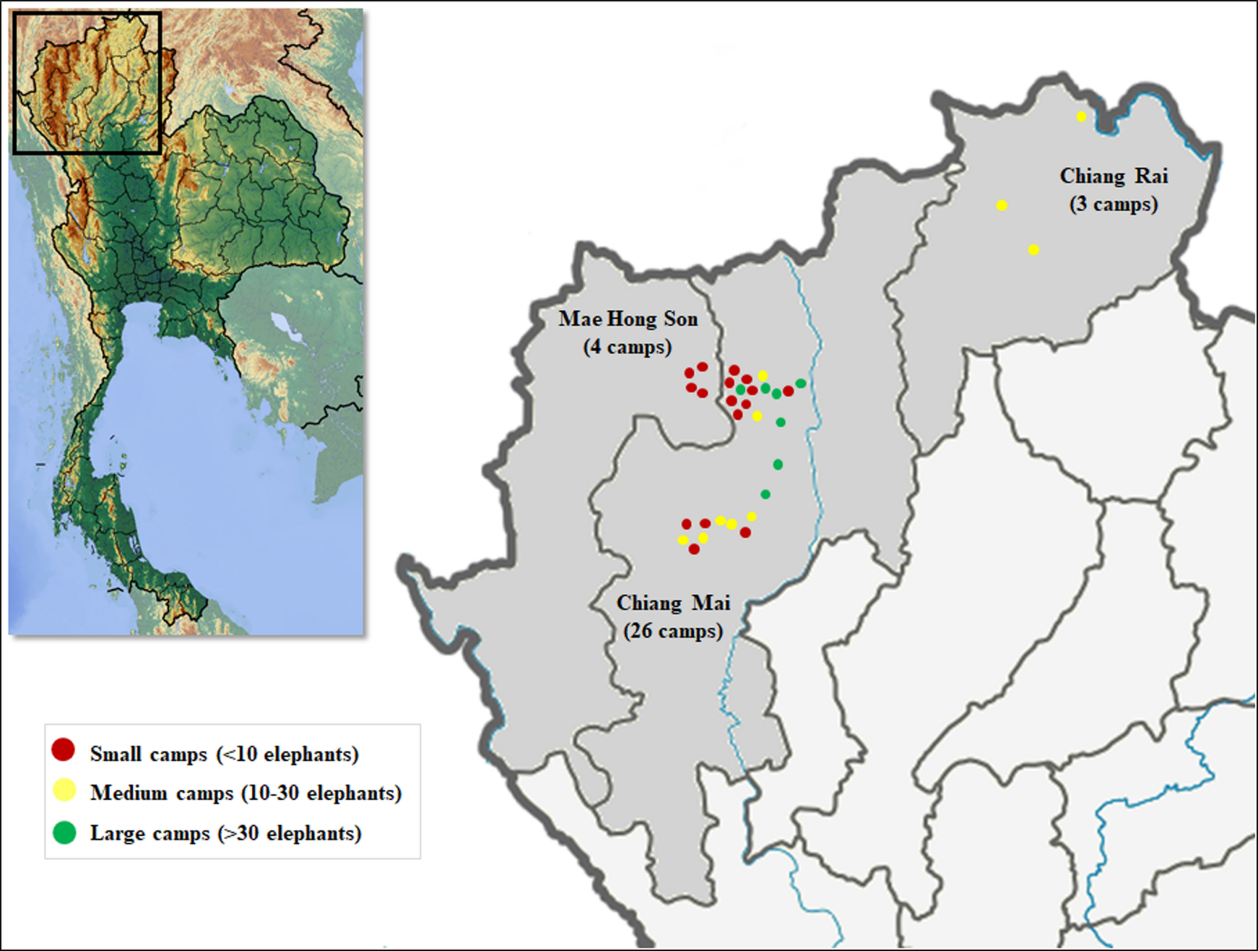
Distribution of elephant camps included in this study by province. The color of the dots represent elephant camp size and corresponding numbers of elephants.

Data collection was carried out from April 2015 to April 2017. A total of 33 camps in three provinces in northern Thailand—Chiang Mai (26 camps), Mae Hong Son (four camps) and Chiang Rai (three camps)—were included in the survey (Fig. 1).

The study consisted of questionnaire interviews (S1) with camp owners, managers, and/or camp veterinarians, and direct observations during camp operating hours. Interviewers and observers were veterinarians experienced in working with elephants from the Veterinary Faculty, Chiang Mai University (CMU). The questionnaire consisted of three sections that took approximately 60–90 min to complete: *Section 1* contained camp and elephant information including location, years of operation, activities, elephant number, sex and age of elephants, and ownership of elephants; *Section 2* covered elephant management related to work activities and hours, equipment, rest areas, chaining, nutrition, health care, and breeding; and *Section 3* consisted of mahout questions related to mahout number, ethnicity, work conditions, salary, benefits, areas for improvement, and rules. Camp activities, facilities and environment were documented based on direct observations, including types of work, walking trails, grass fields, food stores, water sources, rest areas, health records, medicines, and elephant clinics (if present). We defined “clinic” as a separate area in the camp where an elephant can be brought for treatment, and where medicines and health care supplies are stored. Camp mahouts, veterinary assistants, or outside vets can use the clinic.

**Figure 2:**
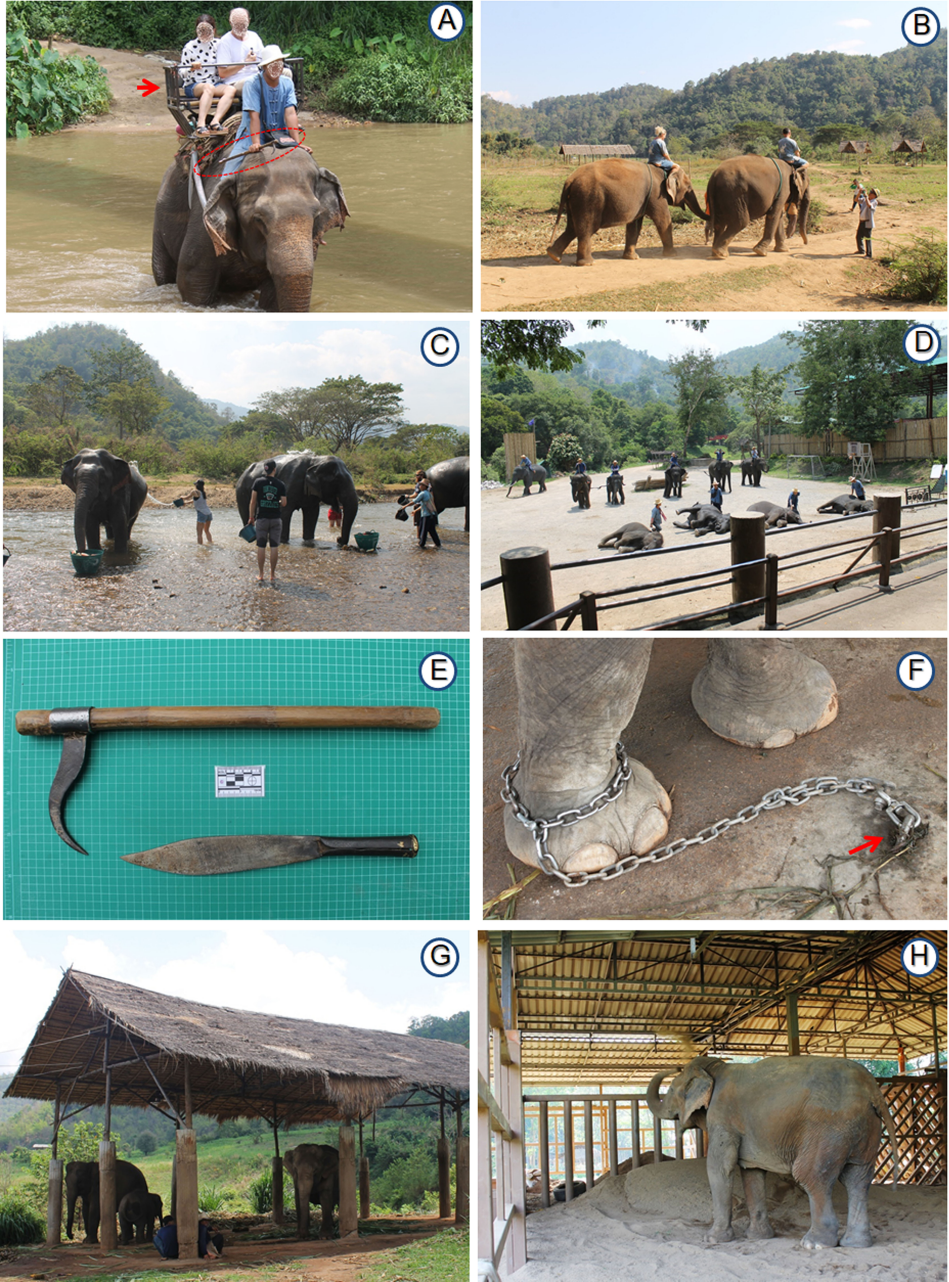
Examples of camp management including tourist activities, housing, and methods of control. (A) A mahout carrying a hook (red circle) during a riding with a saddle (red arrow) program. (B) Riding bareback. (C) No riding, with bathing elephants in a river. (D) Elephant show. (E) Examples of a hook (top) and a knife (bottom) used by mahouts. (F) A pin (red arrow) buried underground to tether a chain. (G) Covered shed with elephants chained near each other. (H) Enclosure to house elephants separately. Photography by Pakkanut Bansiddhi.

Work activities for elephants, or programs with elephants for tourists, were categorized as follows: (a) *Riding with a saddle*: one or two tourists sit on a saddle (seat) with a mahout on the neck (Fig. 2A); (b) *Riding bareback*: one or two tourists sit on the elephant’s neck and/or back, with a mahout following on the trail (Fig. 2B); (c) *No riding*: one or a group of tourists interact with elephants by feeding, bathing and walking on a trail without riding (Fig. 2C); and (d) *Elephant show*: tourists sit and watch a scripted show demonstrating elephant abilities and elephant–mahout interactions (Fig. 2D). In one camp (e), tourists observed the behavior of elephants controlled by mahouts (*Observation*).

### Statistical analysis

Results are presented as percentages, or as the mean ± SE. Chi-square tests of association were used to identify relationships between variables and years of camp operation, camp size, type of work, or location. The variables tested by chi-square tests of association included ownership, sex, type of saddles, removal of saddles, presence of concrete in walking trails and rest areas, use of hooks, presence of roofed structure, floor washing rates, purchasing of roughage, providing free foraging, having veterinarians, having a clinic, and breeding success. Pairwise tests of independence were used as post hoc tests to conduct separate comparisons between variable levels. *P* values from multiple comparisons were adjusted by the Bonferroni’s method.

To compare means of two groups, a *t*-test was used, and a non-parametric Wilcoxon rank sum test was used when the data did not meet either the assumption of normality or the assumption of equal variances. To compare means for more than two groups, one-way analysis of variance was used and a non-parametric Kruskal–Wallis rank sum test was used when the data did not meet either the assumption of normality or the assumption of equal variances. The variables used to compare means included operation hours, walking time and distance, chain length, amount of roughage, and mahout salary. Because there was only one camp in the Observation category, descriptive data were presented, but not used for statistical analyses. Statistical analyses were conducted by using the R program (version 3.4.0; R Core Team, 2014). *P* < 0.05 was considered statistically significant.

## Results

Overall, 33 camps with 627 elephants were surveyed. A total of 79% (*n* = 26) were in Chiang Mai, 12% (*n* = 4) were in Mae Hong Son, and 9% (*n* = 3), were in Chiang Rai provinces. Camps had been in operation from 5 months to 41 years, and were categorized into three groups for analysis: 0–5, 6–15, and >16 years. Camps in operation from 6 to 15 years formed the largest group (43%, *n* = 14), followed by 0–5 (30%, *n* = 10), and >16 (27%, *n* = 9) years. Camps also were divided into three sizes based on elephant numbers: small (<10 elephants; *n* = 16; 49%), medium (10–30 elephants; *n* = 10; 30%), and large (>30 elephants; *n* = 7; 21%). Years of operation was not associated with camp size (χ^2^ = 4.89, *n* = 33, d*f* = 4, *P* = 0.299) (Table 1). Tourist activities other than those involving elephants included riding on an ox-cart, river rafting, hiking, visiting hill tribe villages, and engaging in zip lining.

**Table 1:** Number and percentage (in parentheses) of elephant camps (size of camp) and elephants (for sex) by years of camp operation.

Variable	Years of operation			*P*
	0–5	6–15	>16	
Size of camp				
Small (*n* = 16)	7 (70%)	5 (36%)	4 (45%)	0.299
Medium (*n* = 10)	3 (30%)	5 (36%)	2 (22%)	
Large (*n* = 7)	0 (0%)	4 (28%)	3 (33%)	
Sex				
Male (*n* = 156)	20 (24%)^a^	43 (15%)^b^	93 (37%)^c^	<0.001*
Female (*n* = 471)	63 (76%)^a^	251 (85%)^b^	157 (63%)^c^	

**Notes:**

* Significant at *P* < 0.05 between two variables using chi-square tests of association.

a,b,c Different superscript across rows indicate significant differences for each variable (*P* < 0.05) using pairwise tests of independence.

Camps either owned their own elephants or rented them from others. Some elephants were mahout owned, with both mahouts and elephants paid by the camp in a rental capacity. Other elephants were owned by people that rented them to camps, with the camp being responsible for hiring the mahout. A total of 69% (*n* = 433) of elephants were owned by camps, 23% (*n* = 141) were owned by remote owners, and 8% (*n* = 53) were mahout owned, although the proportions differed based on years of camp operation (χ^2^ = 108.59, *n* = 627, d*f* = 4, *P* < 0.001) and camp size (χ^2^ = 61.53, *n* = 627, d*f* = 4, *P* < 0.001) (Table 2). Newer camps (0–5 years of operation) were significantly more likely to have mahout owned elephants than older camps, while larger camps were more likely to own their own elephants.

**Table 2:** Number and percentage (in parentheses) of elephants for each years of camp operation and size of camp by ownership.

Variable	Elephant *N*	Ownership			*P*
		Camp	Remote owner	Mahout	
Years of operation					
0–5	83	31 (7%)^a^	22 (16%)^b^	30 (57%)^c^	<0.001*
6–15	294	231 (53%)^a^	52 (37%)^b^	11 (21%)^c^	
>16	250	171 (40%)^a^^b^	67 (47%)^a^	12 (22%)^b^	
Size of camp					
Small	90	53 (12%)	26 (18%)	11 (21%)	<0.001*
Medium	145	75 (17%)^a^	39 (28%)^b^	31 (58%)^c^	
Large	392	305 (71%)^a^	76 (54%)^b^	11 (21%)^c^	

**Notes:**

* Significant at *P* < 0.05 between two variables using chi-square test of association.

a,b,c Different superscript across rows indicate significant differences for each variable (*P* < 0.05) using pairwise tests of independence.

### Elephant demographics

**Figure 3:**
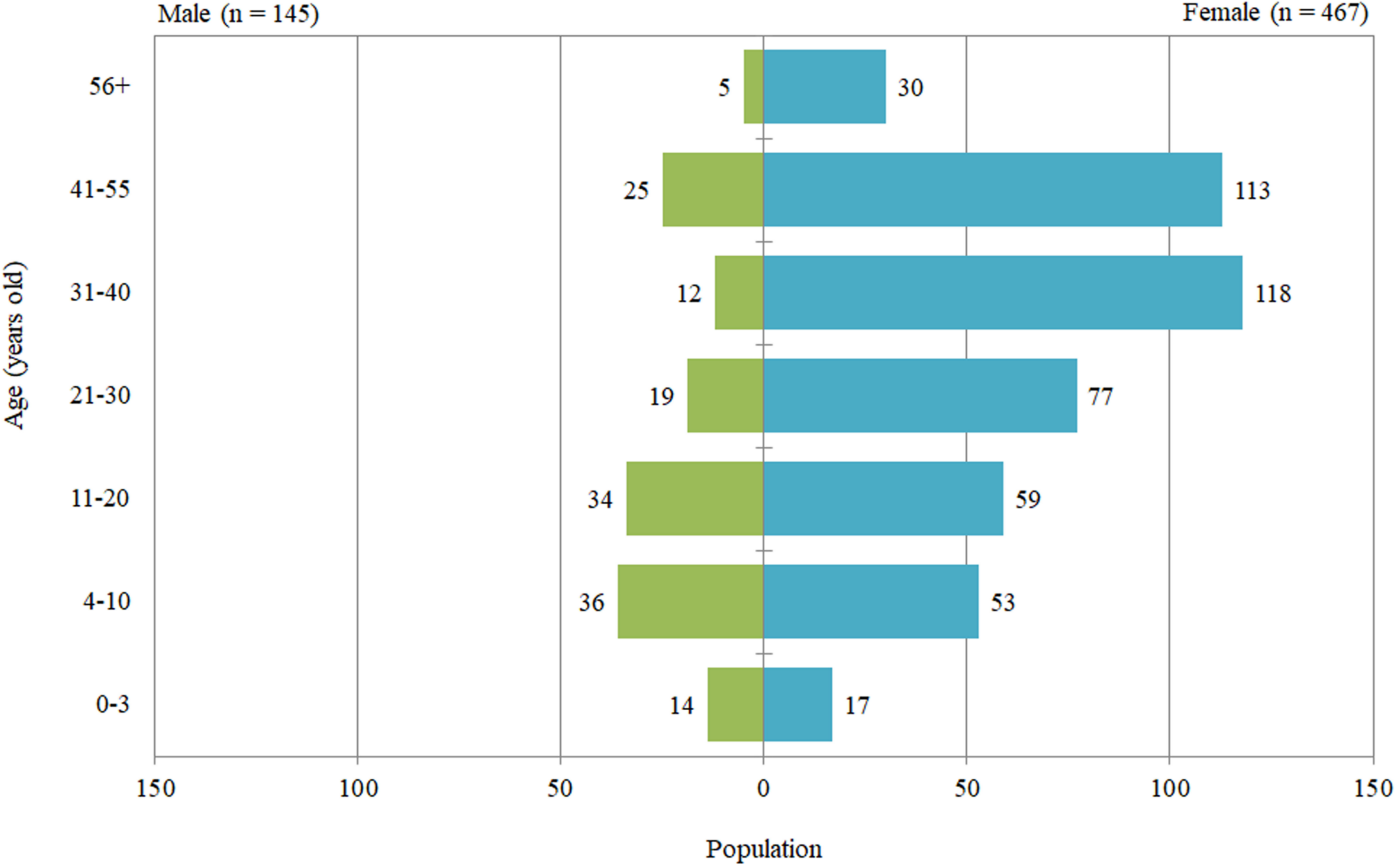
Number of male and female elephants in each age group.

The age for 612 elephants (one camp did not have ages for every elephant) was categorized into seven groups (Fig. 3), with the majority of males being 4–10 years of age, and females being 31–40 years. With both sexes combined, the greatest number of elephants was in the 41–55 year age group. Overall, the ratio of males to females was 1:3, and not related to camp size (χ^2^ = 4.00, *n* = 627, d*f* = 2, *P* = 0.135), but was associated with years of operation (χ^2^ = 36.87, *n* = 627, d*f* = 2, *P* < 0.001) (Table 1), with older camps having more males, and also proportions (1:3.2, 1:5.8, and 1:1.7 for camps in operation 0–5, 6–15, and >16 years, respectively) of males compared to newer camps. A higher proportion of males were 20 years of age or under (58%) compared with females (28%). This resulted in the younger age categories having a sex ratio closer to 1:1 (1:1.2, 1:1.5, 1:1.7) compared to the older age categories (1:4.1–1:9.8) (Fig. 3).

### Work activities

Many camps offered more than one type of work; therefore, camps were categorized into six groups based on main tourist activities (Table 3). A few camps (*n* = 5) offered tourists a choice of riding with a saddle or bareback, which were in the small and medium size categories. Type of work was associated with size of camp (χ^2^ = 17.61, *n* = 32, d*f* = 8, *P* < 0.05) and sex of elephant (χ^2^ = 32.87, *n* = 627, d*f* = 5, *P* < 0.001) (Table 3). A program of riding with a saddle and a show was more likely to be offered by large camps compared to other types of work, and utilized a higher proportion of males (Table 3). Some tourist activities were associated with years of operation (Table 4). Specifically, camps that had been in operation longer were more likely to have a riding with a saddle programs (χ^2^ = 9.02, *n* = 33, d*f* = 2, *P* < 0.05), whereas newer camps were more likely to offer no riding (χ^2^ = 7.11, *n* = 33, d*f* = 2, *P* < 0.05). However, no association were found between years of operation and having a program of riding bareback (χ^2^ = 1.42, *n* = 33, d*f* = 2, *P* = 0.491) or a program of shows (χ^2^ = 3.22, *n* = 33, d*f* = 2, *P* = 0.200).

**Table 3:** Number and percentage (in parentheses) of elephant camps (size of camp) and elephants (for sex) for each size of camp and sex by type of work.

Variable	Type of work						*P*
	Riding with a saddle	Riding with a saddle and show	Riding bareback	Riding with a saddle and riding bareback	No riding	Observation^1^	
Size of camp							
Small (*n* = 16)	4 (57%)	0 (0%)	5 (50%)	3 (60%)	4 (80%)	0	0.024*
Medium (*n* = 10)	3 (43%)	1 (20%)	3 (30%)	2 (40%)	1 (20%)	0	
Large (*n* = 7)	0 (0%)^a^	4 (80%)^b^	2 (20%)^a^^b^	0 (0%)^a^	0 (0%)^a^	1	
Sex							
Male (*n* = 156)	16 (21%)^a^^b^	92 (35%)^a^	33 (22%)^a^^b^	5 (12%)^a^^b^	4 (11%)^a^^b^	6 (9%)^b^	<0.001*
Female (*n* = 471)	59 (79%)^a^^b^	168 (65%)^a^	114 (78%)^a^^b^	37 (88%)^a^^b^	31 (89%)^a^^b^	62 (91%)^b^	

**Notes:**

* Significant at *P* < 0.05 between two variables using chi-square test of association.

a,b Different superscript across rows indicate significant differences for each variable (*P* < 0.05) using pairwise tests of independence.

1 The *Observation* camp was not included in the statistical analysis for size of camp due to *n* = 1.

**Table 4:** Number and percentage (in parentheses) of elephant camps for each type of work by years of camp operation.

Variable	Camp *N*^1^	Years of operation			*P*
		0–5	6–15	>16	
Riding with a saddle					
Yes	17	2 (20%)^a^	7 (50%)^a^^b^	8 (89%)^b^	0.011*
No	16	8 (80%)^a^	7 (50%)^a^^b^	1 (11%)^b^	
Riding bareback					
Yes	15	4 (40%)	8 (57%)	3 (33%)	0.491
No	18	6 (60%)	6 (43%)	6 (67%)	
No riding					
Yes	5	4 (40%)^a^	1 (7%)^a^^b^	0 (0%)^b^	0.029*
No	28	6 (60%)^a^	13 (93%)^a^^b^	9 (100%)^b^	
Show					
Yes	5	1 (10%)	1 (7%)	3 (33%)	0.200
No	28	9 (90%)	13 (93%)	6 (67%)	

**Notes:**

* Significant at *P* < 0.05 between two variables using chi-square test of association.

a,b Different superscript across rows indicate significant differences for each variable (*P* < 0.05) using pairwise tests of independence.

1 Some camps were represented in more than one category if more than one type of work was offered.

For riding bareback, 71% (*n* = 15) of camps tied a cinch around the elephant’s chest for a tourist to hold on to, whereas 29% (*n* = 6) did not use any equipment. Saddles were secured using neck, breast, and tail cinches (Fig. 2A). Layers of saddle pads (e.g., gunnysacks, hammered bark of patana oak (*Careya arborea*), blankets, and sponge material) were used as a barrier between the saddle and the elephant’s skin. The weight of the saddle pad was about 50 kg. Saddles were made of wood (∼25 kg) or steel (∼15 kg). A total of 78% (*n* = 14) of riding camps used steel saddles only, 17% (*n* = 3) used wooden saddles only, and 5% (*n* = 1) used both. Type of saddle used was not associated with camp years of operation (χ^2^ = 2.46, *n* = 18, d*f* = 4, *P* = 0.651) or size (χ^2^ = 9.16, *n* = 18, d*f* = 4, *P* = 0.057). A total of 61% (*n* = 11) of those camps took the saddle off the elephant’s back during break time at noon or when there was no work. Removal of the saddle during breaks was not associated with years of camp operation (χ^2^ = 1.70, *n* = 18, d*f* = 2, *P* = 0.429) or size (χ^2^ = 1.17, *n* = 18, d*f* = 2, *P* = 0.557). For camps that had a show, activities performed by elephants included painting (100%, *n* = 6 camps), dancing (67%, *n* = 4), kicking footballs (67%, *n* = 4), lifting up legs (67%, *n* = 4), lying on side (67%, *n* = 4), playing musical instruments (50%, *n* = 3), playing with a hula hoop (50%, *n* = 3), giving massages to its mahout (50%, *n* = 3), demonstrating logging skills (50%, *n* = 3), sitting up (33%, *n* = 2), standing on hind legs (33%, *n* = 2), walking on hind legs (17%, *n* = 1), playing golf (17%, *n* = 1), throwing darts (17%, *n* = 1), and riding a bicycle (17%, *n* = 1).

Average amount of time camps were open (i.e., operation hours) was about 7 h per day, calculated from the time elephants were brought to work until they returned to the night resting area. There were differences in mean operating hours among camps, with those in Chiang Rai and Mae Hong Son open 2.5–3 h longer than those in Chiang Mai (*F*_2,30_ = 12.91, *P* < 0.001) (Table 5). There were no differences in mean operating hours based on years of camp operation (*F*_2,30_ = 1.22, *P* = 0.309) or size (*F*_2,30_ = 0.04, *P* = 0.961). The five camps that offered both riding with a saddle and riding bareback options (small and medium size camps) were open longer than camps offering a range of other activities (*F*_4,27_ = 5.66, *P* < 0.01) (Tables 3 and 5). There were regular days off for elephants in 12% (*n* = 4) of camps, usually 2–4 days per month. Bulls in musth were removed from tourist areas, isolated, and not used for work until they were no longer in musth. If pregnancy was suspected, daily work load was typically reduced in the second year, and stopped altogether 1–3 months before expected parturition. Work load and type of work were modified in old elephants in most of those camps, for example, reduced walking rounds, keeping in a separate geriatric camp, or no longer worked at all.

**Table 5:** Range and mean (±SE) working hours of elephant camps in each location, and based on years of camp operation, size of camp, and type of work.

Variable	Camp *N*	Time work start	Time work end	Mean working hours
Location				
Chiang Mai	26	07:00–10:30	12:00–17:00	6.06 ± 0.3^a^
Chiang Rai	3	07:00–08:00	15:30–17:00	8.50 ± 0.3^b^
Mae Hong Son	4	07:00–09:00	17:00	9.25 ± 0.5^b^
Years of operation				
0–5	10	07:30–10:30	15:00–17:00	6.65 ± 0.5
6–15	14	07:00–10:30	12:00–17:00	6.21 ± 1.1
>16	9	07:00–09:30	14:30–17:00	7.39 ± 0.3
Size of camp				
Small	16	07:00–10:30	14:00–17:00	6.63 ± 0.5
Medium	10	07:00–10:00	12:00–17:00	6.80 ± 0.6
Large	7	07:00–10:00	14:30–15:00	6.57 ± 0.4
Type of work				
Riding with a saddle	7	08:00–10:00	12:00–16:00	6.00 ± 0.7^a^
Riding with a saddle and show	5	07:00–09:00	14:30–17:00	7.50 ± 0.5^a^^b^
Riding bareback	10	07:00–10:30	14:30–17:00	6.20 ± 0.4^a^
Riding with a saddle and riding bareback	5	07:00–09:00	15:30–17:00	9.10 ± 0.4^b^
No riding	5	07:00–10:30	14:00–16:30	5.50 ± 0.7^a^
Observation	1	09:30	15:00	5.50

**Note:**

a,b Different letters across columns indicate significant differences for each variable (*P* < 0.05).

Average walking distance of elephants was 4.5 km per day, with a mean walking time of 158 min per day (Table 6). There were no differences in walking distance (Kruskal–Wallis chi-squared = 1.31, *n* = 44, d*f* = 2, *P* = 0.521) or walking time (Kruskal–Wallis chi-squared = 2.39, *n* = 50, d*f* = 3, *P* = 0.496) based on work type. Walking routes consisted of at least some dirt (100% of camps), with 58% (*n* =19) of camps having at least some portion of the walking path constructed of concrete. The presence of concrete walking trails was associated with years of camp operation, and were more prevalent in older camps (χ^2^ = 6.73, *n* = 33, d*f* = 2, *P* < 0.05). In the one *Observation* camp, total walking distance was estimated to be <2 km per day; elephants were walked around a limited space of natural substrate by the mahouts during tourist hours, and brought to a nearby river to drink and bathe.

**Table 6:** Summary of work activities (mean ± SE) for elephants used in riding, walking/bathing, or shows. Each round represents one completed or discrete tourist-related activity.

Type of work	Camp *N*	Times per day (round)	Distance per round (m)	Time per round (min)	Total distance per day (m)	Total time per day (min)
Riding with a saddle	18	5 ± 0.5	1,043 ± 216.3	35 ± 3.6	4,704 ± 841.7	158 ± 17.2
Riding bareback	21	3 ± 0.5	1,716 ± 277.0	54 ± 8.6	4,991 ± 965.2	158 ± 27.1
No riding	10	2 ± 0.3	1,441 ± 392.6	68 ± 17.4	3,258 ± 1044.7	146 ± 34.2
Show	6	4 ± 1.0		44 ± 6.3		190 ± 17.32

When working, elephants were controlled by the mahout using verbal commands, and equipment that included hooks (85%, *n* = 28 camps) (Fig. 2E), knives (55%, *n* = 18), nails (18%, *n* = 6), slingshots (18%, *n* = 6), and chains that went from the leg to around the elephants’ neck (42%, *n* = 14). Knives were primarily used to cut grass or food for elephants, but also to control elephants, if necessary. A total of 76% (*n* = 25) of camps allowed every mahout to carry a hook, 9% (*n* = 3) allowed hooks only for mahouts who controlled aggressive elephants, and 15% (*n* = 5) did not allow hooks. A hook was carried in the hand or kept in a bag. Use of hooks was not associated with years of camp operation (χ^2^ = 0.26, *n* = 33, d*f* = 2, *P* = 0.878) or work type (χ^2^ = 5.92, *n* = 32, d*f* = 4, *P* = 0.205). For the camps that did not allow hooks (*n* = 5), one camp used nails and slingshots instead, the other camp used a chain around the elephant’s neck. Three camps did not allow mahouts to carry any equipment: a *No riding* camp with four female elephants; a *No riding* camp with five older female elephants (>55 years of age); and the *Observation* camp (but see Discussion). A total of 88% (*n* = 29) of camps reported injuries in elephants, caused by restraint equipment (67%, *n* = 22), saddle equipment (33%, *n* = 11), improper walking trails (42%, *n* = 14), or intra-species aggression (52%, *n* = 17).

### Housing and rest areas

A total of 88% (*n* = 29) of camps offered one or more roofed structures to shelter elephants from the elements. Of these, 52% (*n* = 15) had enough covered space to accommodate every elephant, whereas 12% (*n* = 4) provided no roofed structures. There were two main types of covered structures: sheds (Fig. 2G) and enclosures (Fig. 2H). Sheds were normally used to keep several elephants in the same place that were separated using ropes or chains. Enclosures housed one or two elephants that were either tied to an internal post or allowed free movement; enclosure sizes ranged from 6 by 6 to 12 by 15 m. For camps that offered covered shelters, 69% (*n* = 20) had only sheds, 14% (*n* = 4) had only enclosures, and 17% (*n* = 5) had both. Whether a camp had a roofed structure or not was not related to years of camp operation (χ^2^ = 4.57, *n* = 33, d*f* = 2, *P* = 0.102) or size (χ^2^ = 2.07, *n* = 33, d*f* = 2, *P* = 0.355).

When not working during the day, 30% (*n* = 10) of camps kept elephants under a roofed structure, 3% (*n* = 1) secured elephants under a tree for shade, 12% (*n* = 4) provided no shade (open areas, grass fields), 6% (*n* = 2) separated elephants between tree-shaded and no shaded areas, and 49% (*n* = 16) separated elephants between roofed and no-roofed conditions. At night, 34% (*n* = 11) of camps kept elephants under a roofed structure, 3% (*n* = 1) secured elephants in a forested area on a long chain, 12% (*n* = 4) provided no cover (open areas, grass field), 21% (*n* = 7) alternated elephants between a forested area and area with no cover, and 30% (*n* = 10) separated elephants using both roofed and no-roofed conditions. There were four camps that kept elephants under a roofed structure when not working both day and night.

When not working, 82% (*n* = 27) of camps chained elephants at least some of the time (Fig. 2F); 9% (*n* = 3) chained elephants only a night, and 9% (*n* = 3) did not chain elephants at all. Chains at night were on average twice as long as those used during the day (*W* = 251.50, *n* = 27, *P* < 0.05) (Table 7). In camps that did not use chains, elephants were controlled by mahouts during the day, and kept in enclosures at night. For camps that used chains, elephants were tethered to pins (Fig. 2F) (83%, *n* = 25 camps), poles (13%, *n* = 4), trees (10%, *n* = 3), or the chains were buried in the ground (3%, *n* = 1). A total of 58% (*n* = 19) of camps reported experience with controlling musth bulls, which were kept separated and in specific areas away from tourists on short (2–5 m; *n* = 12; 63%) or long (10–20 m; *n* = 6; 32%) chains. One camp kept musth bulls in covered enclosures. A total of 58% of these camps (*n* = 11) had experienced mahouts to control musth bulls. A total of 26% (*n* = 5) of these had a dart gun with immobilizing drugs, and 11% (*n* = 2) conducted emergency drills related to musth bull control (all large camps).

**Table 7:** Mean (±SE) chain length for tourist camps that chain elephants during the day or night time.

Chain period	Camp *N*	Chain length	Chain length	*P*
		Day (m)	Night (m)	
Chain only at night	3	−	9.00 ± 5.6	−
Chain both day and night	27	2.94 ± 0.7	5.87 ± 1.4	0.049*

**Note:**

* Significant using Wilcoxon rank sum test.

Three types of flooring were used in the rest areas: natural substrate (primarily dirt); concrete; and sand. A total of 52% (*n* = 17) of camps housed elephants on both dirt and concrete floors (e.g., concrete floors during the day and dirt floors at night), 45% (*n* = 15) provided dirt floors only, and one camp had elephants on natural substrates during the day and sand stalls at night. There were no camps that maintained elephants on concrete all of the time. Presence of concrete in rest areas during daytime was not related to years of camp operation (χ^2^ = 1.45, *n* = 33, d*f* = 2, *P* = 0.484) or camp size (χ^2^ = 2.43, *n* = 33, d*f* = 2, *P* = 0.296). Presence of concrete in rest areas during nighttime was not related to years of camp operation (χ^2^ = 1.83, *n* = 33, d*f* = 2, *P* = 0.401) or camp size (χ^2^ = 4.35, *n* = 33, d*f* = 2, *P* = 0.114). Dirt floors were normally cleaned daily by removing feces and discarded food. For camps that provided concrete floors, the floors were washed daily (53%, *n* = 9 camps), weekly (18%, *n* = 3), or monthly (11%, *n* = 2). Three camps did not wash concrete floors at all. Overall, washing rates were not associated with years of camp operation (χ^2^ = 2.53, *n* = 17, d*f* = 6, *P* = 0.866) or size (χ^2^ = 9.44, *n* = 17, d*f* = 6, *P* = 0.150).

### Nutrition

The mean number of feedings was three times daily: early morning, midday, and evening/night. Fresh roughage was the main food source, and included corn stalks (85%, *n* = 28 camps), napier grass (*Pennisetum purpureum*) (61%, *n* = 20), bana grass (a hybrid derived from pearl millet (*P. americanum*) and napier grass) (39%, *n* = 13), pineapple stalk (15%, *n* = 5), banana trunk (12%, *n* = 4), bamboo grass (12%, *n* = 4), and grass hay (9%, *n* = 3). A total of 9% (*n* = 3) of camps regularly fed one variety of roughage, 49% (*n* = 16) fed two varieties, 36% (*n* = 12) fed three varieties, 3% (*n* = 1) fed four varieties, and 3% (*n* = 1) fed five varieties. A total of 24% (*n* = 8) of camps purchased all of their roughage, 12% (*n* = 4) grew their own, and 64% (*n* = 21) did both. Whether a camp purchased roughage or not was not related to years of camp operation (χ^2^ = 5.78, *n* = 33, d*f* = 2, *P* = 0.056) or size (χ^2^ = 1.67, *n* = 33, d*f* = 2, *P* = 0.434) (Table S1). The amount of food offered was based in part on sex, age, and body size of elephants, but was not precisely calculated. Mean estimated amount of roughage fed was 125 kg per day in adult females and 116 kg per day in adult males. There were no differences in amount of roughage provided to females (Kruskal–Wallis chi-squared = 3.34, *n* = 28, d*f* = 2, *P* = 0.188) or males (Kruskal–Wallis chi-squared = 4.68, *n* = 19, d*f* = 2, *P* = 0.096) based on camp size. In camps with both adult males and females, elephants were fed the same amount of roughage, except one camp that fed adult males more than females. Elephants that had a larger body size were generally fed a greater amount of food. The highest amount fed was 300 kg, and the lowest was 20 kg. A total of 39% (*n* = 13) of camps tethered elephants in a nearby forest or grass field for free foraging, but this activity did not affect the reported amount of roughage provided to females (*t* = 0.85, d*f* = 26, *P* = 0.401) or males (*t* = 1.19, d*f* = 17, *P* = 0.252). However, there were two camps that provided less than 60 kg of roughage per day, both of which provided free foraging. Providing free foraging opportunities was associated with camp years of operation (χ^2^ = 9.91, *n* = 33, d*f* = 2, *P* < 0.01) (Table S2), and more common in the newer camps.

Camps also fed elephants a number of supplements. All of the surveyed camps fed bananas, which were most often offered by tourists. Other supplements included sugarcane (*Saccharum officinarum*) (88%, *n* = 29 camps), tamarind (*Tamarindus indica*) (42%, *n* = 14), commercial pellet feed (27%, *n* = 9), watermelon (21%, *n* = 7), pumpkin (21%, *n* = 7), sticky rice (18%, *n* = 6), paddy (15%, *n* = 5), pineapple (9%, *n* = 3), and cucumber (9%, *n* = 3). Elephants were fed supplements by mahouts and/or tourists during the day in various amounts. Herbal supplements were fed regularly in a few camps (18%, *n* = 6) as another source of nourishment. Nutrition management was often modified in pregnant, old, sick, or musth bull elephants. For example, food was chopped to small pieces for old elephants (33% of camps with old elephants; *n* = 4), or high energetic food intake was reduced for musth bulls (84% of camps with musth bulls; *n* = 16).

Drinking frequency of elephants was about three times per day (early morning, midday, and evening/night) or depended on their mahouts. The last time that elephants were provided water by mahouts was about 17:00–19:00 h. During nighttime, 82% (*n* = 27) of camps had no water sources in rest areas for elephants, 15% (*n* = 5) had water sources for some elephants, and one camp had water sources for every elephant. The most common sources of water for bathing and drinking were nearby rivers or streams. In many camps, clean water was piped from a mountain stream to the camps. In Mae Hong Son, elephants were provided mineral water piped in from a local natural hot spring.

### Health care

A total of 18% (*n* = 6) of camps had resident veterinarians, and 36% (*n* = 12) had an elephant clinic. Large camps were more likely to have onsite veterinarians (χ^2^ = 13.46, *n* = 33, d*f* = 2, *P* < 0.01) (Table S3) and a clinic (χ^2^ = 16.36, *n* = 33, d*f* = 2, *P* < 0.001) (Table S4). There were no association between years of operation and having veterinarians (χ^2^ = 4.46, *n* = 33, d*f* = 2, *P* = 0.108) (Table S3) or a clinic (χ^2^ = 4.69, *n* = 33, d*f* = 2, *P* = 0.096) (Table S4), however. A total of 61% (*n* = 20) of camps had staff who passed a veterinary assistant training course held by the Thai Elephant Conservation Center (TECC) in Lampang province, or the Center of Excellence in Elephant and Wildlife Research, CMU. A number of camps that did not have their own veterinary staff (39%, *n* = 13) relied on veterinarians from nearby camps, the TECC, CMU or a local livestock office within the DLD. For severe or complicated cases, camps sent their elephants to hospitals at the TECC or Friends of the Asian Elephant Foundation for treatment. All camps were visited twice a year by veterinarians from the TECC, CMU, or the DLD National Institute of Elephant Research and Health Service, who conducted routine health exams and provided deworming services.

### Breeding programs

A total of 49% (*n* = 16) of camps had both bulls and cows of breeding age, 42% (*n* = 14) had only cows of breeding age, and 9% (*n* = 3) did not have breeding age elephants. A total of 21% (*n* = 7) of all camps were not actively involved in a breeding program, although they had breeding age elephants. Five of these camps had cows with no bulls, while two chose not to breed to control their population. A total of 61% (*n* = 20) of camps took elephants to other camps for breeding and/or had elephants from other camps come to their camp for breeding. In Mae Hong Son province, there were no breeding bulls in any of the camps.

For camps that had breeding females, 83% (*n* = 25) actively detected estrus. Of those, 77% (*n* = 23) were based on mahout observations (i.e., discharge from vulva, behavioral changes), 53% (*n* = 16) exposed females regularly to a breeding bull for genital examination, and 30% (*n* = 9) analyzed serum progestagens using the endocrine laboratory at CMU. Females considered to be in estrus were generally taken to a quiet place away from tourists for breeding under mahout observation. However, breeding was not prevented in tourist areas, and sometimes occurred. Average age of first breeding was 7.5 years old (range, 7–20 years). A total of 55% (*n* = 15) of camps reported successful births over the past 5 years. Breeding success were better in larger camps (χ^2^ = 9.46, *n* = 33, d*f* = 2, *P* < 0.01) and those in operation longer (χ^2^ = 7.31, *n* = 33, d*f* = 2, *P* < 0.05) (Table S5), as determined by the number of calves born at the camp in the last 5 years.

### Mahout management

All camps employed one mahout per elephant. A total of 12% (*n* = 4) of camps used Thai mahouts only, 88% (*n* = 29) used mahouts who were either Thai or one of several ethnic groups, including Karen, Tai Yai, and Palaung. Only 15% (*n* = 5) of camps offered regular days off for mahouts, normally two to three consecutive days per month, whereas 85% (*n* = 28) did not. Mean mahout salary was 7,846 baht per month (range, 5,000–11,500 baht), and was not related to years of camp operation (*F*_2,23_ = 2.71, *P* = 0.088), size (*F*_2,23_ = 2.06, *P* = 0.151) or the type of work (*F*_4,20_ = 1.33, *P* = 0.292) (Table S6). In many camps, mahouts received extra income through tips from tourists (67%, *n* = 22 camps), doing extra rounds of riding activities (30%, *n* = 10), or by selling wood carvings (*n* = 1). Some camps provided mahouts with extra benefits, such as free housing (39%, *n* = 13), rice (9%, *n* = 3), and other meals (12%, *n* = 4). Few camps (*n* = 3) offered health programs or insurance for their mahouts. A total of 88% (*n* = 29) of camps had sent their mahouts to mahout training courses to improve skills in elephant training and/or health care. Camps had rules for mahout behavior, such as properly using tools and only in case of necessity (67%, *n* = 22 camps), no use of alcohol during working hours (45%, *n* = 15), and prohibition of narcotics (24%, *n* = 8). All camps indicated there were punishments for mahouts that broke the rules, including warnings, suspension of work, deductions in pay, transfers to other positions, or termination.

## Discussion

This study represents the first survey of activities and management practices of tourist-oriented camps in northern Thailand in nearly a decade, and the most comprehensive in scope to date. The study population represented 71% of the total elephant population in the region, and 100% of large, 50% of medium, and 38% of small camps in the region. Data reveal considerable variation in captive elephant demographics, tourist activities, health care, and mahout management among camps, with some major differences observed between older established and newer, recently established camps. Newer camps were more likely to have elephants owned by mahouts. From the interview, we found that many new camp owners were mahouts that had previously worked in larger camps, and had the expertise to establish their own business. Often, these camps relied on the help of both immediate and extended family members. In the last survey, popular tourist activities included elephant feeding, riding with a saddle, and a show (Kontogeorgopoulos, 2009a, 2009b), which was the case for many of the camps in this study. Older camps were more likely to have a program of riding with a saddle, involving walking short distances around the camp area or in local village areas, with an increased presence of concrete on the walking paths. Perhaps in response to pressure by animal activists, and travel agency hesitance to book elephant-riding in recent years (Center for Responsible Travel, 2016), many newer camps offered no riding programs, and were more likely to provide free-foraging in local forest areas for the elephants. Thus, it appears new camp owners may be more responsive to welfare concerns, and need for elephants to express natural behaviors and forage for a variety of foodstuffs. Captive elephants still need to be controlled, however, whether they are at camps with no riding programs or are used in more intensive activities. This was confirmed by the finding that use of hooks was not associated with years of camp operation or the type of work elephants were involved in. However, new camps were less likely, or perhaps less willing, to have bull elephants on site because of the increased danger they pose because of musth, a temporary state of heightened aggressive and unpredictable behavior in association with elevated testosterone (Duer, Tomasi & Abramson, 2016). Overall, compared to earlier surveys, elephant management practices appear to have improved, which could signal a positive trend toward improving animal care. The next step will be to determine how various management practices affect biological and behavioral outcome measures, something that is necessary before declaring what practices are good or bad for welfare.

### Elephant demographics

In the 2009 survey of elephant camps in northern Thailand, Godfrey & Kongmuang (2009) reported most elephants were in the 31–40 year age group, which is in accordance with our finding that nearly 10 years later, the maximum elephant numbers were in the 41–55 year age group. In another 10 years, these elephants will be of geriatric age, which means it will be necessary to develop strategies to effectively manage specific health and nutritional needs. There was one camp in this study that established a separate geriatric camp where old elephants received special diets, herbal treatments, and allowed tourists to observe and learn, something other camps may want to consider going forward.

Our finding of a sex ratio of 1:3 (male:female) differed from that of earlier reports of a 1:2 sex ratio in northern Thailand (Godfrey & Kongmuang, 2009) and throughout Thailand (Pintavongs et al., 2014). The sex ratio observed by Schmidt-Burbach, Ronfot & Srisangiam (2015) in north, central and south Thailand tourist camps was 1:4. Differences may reflect the trend toward newer camps obtaining primarily females, which generally are less aggressive, and thus better suited for interacting with tourists under more natural conditions. In Myanmar timber elephants, male calves had higher mortality, possibly linked to higher parasite loads, infection rates, accidents and injuries during breaking after weaning (Mar, Lahdenperä & Lummaa, 2012). The lack of a comprehensive record keeping system in Thailand precludes determining if there is a similar sex bias in calf mortality. Under more natural conditions, like a semi-captive population at the Pinnawala Elephant Orphanage in Sri Lanka (Pushpakumara et al., 2016) or in the wild (Sukumar, Ramakrishnan & Santosh, 1998), the sex ratio at birth is closer to 1:1. An interesting finding was that the sex ratios differed across age categories. A higher proportion of males were 20 years of age or under (54%) compared with females (27%), which resulted in the younger age categories having a sex ratio closer to 1:1 (1:1.2, 1:1.5, 1:1.7) compared to the older age categories (1:4.1–1:9.8). As more camps become involved in breeding, which currently is more common in the older camps, the sex ratio is likely to continue to shift to a more equal distribution of males and females across age groups. This change will present new challenges for ensuring good welfare for bulls, which can be difficult to manage and socialize, and often are isolated or kept on short chains for prolonged periods of time, especially during musth, as a safety precaution.

### Work activities

It is important to note that operating hours were from camp opening to closing, which refer to the total time elephants were under the control of mahouts and had the potential to interact with tourists. Therefore, operating hours did not reflect actual working hours of elephants. Elephants generally were given rest periods between working rounds, when there were no tourists, and at noon (about 1 h). During these rest periods, elephants often foraged or were offered browse and sometimes water. Besides operating hours, we included questions about walking distances to understand work intensity of elephants. Walking distances have been reported to range from 3.2 to 8.9 km per day in wild Asian elephants (Rowell, 2014), seven km per day in forest camps of India (Varma et al., 2010), and 5.3 km per day in North American zoos (Holdgate et al., 2016b). Thus, the mean walking distance of 4.5 km per day in this study was comparable. From a welfare standpoint, Holdgate et al. (2016b) and Miller, Hogan & Meehan (2016) found walking distance was not related to health or behavioral outcomes (i.e., foot, joint, body condition, stereotypy); however, Morfeld et al. (2016) demonstrated that walking exercise of 14 h or more per week reduced the risk of being overweight or very overweight. However, those studies focused on zoo elephants, which were managed under different environment and management conditions compared to captive elephants in Asia. We did not measure walking pace in this study; however, walking at a faster than normal pace has been associated with signs of fatigue (i.e., sleepy daze, stupor, or trance) in circus elephants (Ensley, 2012).

Most elephant camps in this study reported using equipment, particularly hooks (85% of camps), to control elephants. Grandin (1999) acknowledged there are certain inherent dangers when working with large animals, and training animals to cooperate with handling procedures helps reduce stress and accidents. Elephant body regions have different sensitivities and therefore require different pressures to induce desired responses. The intended purpose of the hook is not to cause pain or to punish, but to apply pressure to particular control points that the elephant has been trained to react to: stop, turn left, turn right, kneel, stand still and others (Phuangkum, Lair & Angkawanith, 2005). However, the forceful or inappropriate use of a mahout’s hook can cause painful puncture wounds, which is a welfare concern (Clubb & Mason, 2002; Kontogeorgopoulos, 2009a). In this study, 67% of camps reported injuries caused by restraint equipment, mainly hooks. However, about half of camps (52%) reported injuries due to fighting between elephants, both related and unrelated, which might be from establishing social hierarchies when housed in confined spaces. This situation confirms the importance of being able to properly control elephants in free contact situations to mitigate injuries. Camps are aware of problems with misuse of the hook, as over two thirds reported mahouts receive verbal instructions on proper equipment use. There also are written guidelines for the proper use of hooks in Thailand (Phuangkum, Lair & Angkawanith, 2005).

A total of 15% of camps did not allow mahouts to carry hooks, presumably in response to tourists’ concerns about using equipment to control elephants, and the presumption that such tools are always cruel (Schmidt-Burbach, 2017; World Animal Protection (WAP), 2014). At some camps where no hooks were used, mahouts sometimes carried nails in their pockets or used slingshots for protection. Nails actually present a higher risk because the elephant has no visual cue, and a mahout has to be in much closer contact to use them. Slingshots can cause injuries to eyes and nails, and require skill to use. There was no guarantee that every camp answered honestly about equipment use, and although observers were able to see hooks, knifes, and chains, it was not possible to identify small items in pockets, like nails and slingshots. It may not always be possible to ensure that an elephant can be safely controlled at all times, and will not present a danger to tourists, mahouts or other elephants in a free contact situation without such tools. A better and more honest approach would be for camps to explain to tourists the importance of and the principles behind using a hook, and that carrying a hook is often necessary for the safety of elephants and people alike.

We found a variety of work equipment and activities across camps, such as type and weight of saddles, type and weight of saddle padding, and type of activities highlighted in the shows, all of which can affect elephants physically, although few studies have investigated these specific aspects of elephant welfare. Khawnual & Clarke (2002) reported the presence of abrasions caused by the movement of a saddle and girth strap. Similarly, Magda et al. (2015) found a high prevalence of lesions related to saddle equipment, most often located on the back region. The probability of an active lesion increased with the number of working hours per day with a peak at 6–7 h, and was related to the age of the elephant. In this study, more than half of camps that provided riding with a saddle program (61%) reported injuries caused by saddle equipment, thus representing a welfare concern. Lesions result primarily from ill-fitting saddles or inadequate or inappropriate padding material. In particular, use of rice sacks cause more lesions than other materials, such as bark, carpet or blankets (Magda et al., 2015). In our study, camps generally used gunnysacks, hammered bark, blankets, or sponge material as saddle padding, yet there still were incidences of injuries. Shape of the backbone can also be a factor, with higher ridgelines being more susceptible to saddle injuries, although this has yet to be studied in detail. In circus elephants, arthritis, lameness, joint problems, as well as hernias, are thought to result from elephants repeatedly assuming unnatural positions during performances (Wilson, 2015). Kuntze (1989) also claimed that the sitting position can lead to serious problems and even death, if the intestines, uterus or bladder prolapses or becomes constricted and necrotic. For camps that had a show, a number of them reported activities that included sitting up (33%), standing on hind legs (33%), walking on hind legs (17%), and riding a bicycle (17%), which could cause similar problems. More research is needed to determine the effects of each work type on health and welfare, and how camps can minimize work-related injuries.

### Housing and rest areas

Approximately one third of the camps provided shade to all of their elephants during the day if needed, either as a covered structure or securing to a tree, while 12% offered no protection from the elements at all. Provisioning of roofed or shaded structures is likely related to financial commitments to infrastructure by the camps, something that has been shown to be related to providing good health care and diets (Kontogeorgopoulos, 2009a). According to guidelines for western zoo elephants, both indoor and outdoor spaces should be provided for elephants (Association of Zoos and Aquariums (AZA), 2012; British and Irish Association of Zoos and Aquariums (BIAZA), 2010). In elephant camps, daytime shelters are critical to provide protection from sun and rain during working hours (Phuangkum, Lair & Angkawanith, 2005). Elephants exposed to hot direct sunlight for extended periods of time can become sunburned and may suffer heat stroke (Adams, 1981; Fowler, 2008). In addition, shelter may become an important factor in the winter when temperatures decline and some elephants actively shiver (Varma & Ganguly, 2011). Therefore, each elephant must have protection from the elements, and if camps have limited budgets, they could at least provide temporary shelter using inexpensive materials, such as grass or banana tree leaves to make a roof (Phuangkum, Lair & Angkawanith, 2005).

Elephants being restrained by chains is a topic of intense debate. For most facilities, it is not financially or logistically feasible to build reinforced enclosures for restraint. Rather, chaining is the easiest and cheapest way to control several elephants in the limited space of an elephant camp. However, chaining for prolonged periods limits their movement, may cause joint damage or skin abrasions, and have negative effects on foot health (Buckley, 2008; Mikota, 2008). Moreover, studies have shown that chaining is associated with stereotypic behaviors, as in circus elephants where the probability of stereotypic behaviors was higher when leg-chained (Gruber et al., 2000). Greco et al. (2017) also reported chain chewing (taking a chain into the mouth and biting or sucking on it) as another type of stereotype behavior found in confined elephants. In southern India, Varadharajan, Krishnamoorthy & Nagarajan (2016) found a higher prevalence of stereotypies in Hindu temples (49%) and private elephants (25%) chained an average of 20 and 18 h per day, respectively, compared to those chained by the Forest Department about 6 h per day (7%). In western zoos, chaining is acceptable during medical treatments or other short-term interventions, but not for prolonged restraint (British and Irish Association of Zoos and Aquariums (BIAZA), 2010). We did not specifically measure the amount of time each elephant was on chains, but in general most were restrained in the afternoon/evening after work until the next morning, and when they were not working during the daytime.

According to the Elephant Care Manual for Mahouts and Camp Managers published a decade ago by the Food and Agriculture Organization of the United Nations, adult elephants in natural or open environments should be kept on chains between 20 and 30 m in length (Phuangkum, Lair & Angkawanith, 2005), although it was recognized that shorter chains might be necessary in confined areas. Today, this recommendation no longer applies to the vast majority of camps in Thailand due to restricted space, and in our study, average chain length was three m during the day and six m at night. Minimum chain lengths or duration of chaining are not specified in any of the welfare standards in Thailand, so camps are free to make those decisions without regard to elephant needs. The finding that 82% of camps chained elephants for a significant part of the day leads us to conclude it is an important area in need of improvement, and a welfare concern if elephants are kept socially isolated and restricted in movement for too long. In general, chains were used in large, free contact situations and for controlling adult males. There were six camps that did not use chains during the day and relied on the constant presence of a mahout. Four of these had only females, one had two young males (less than 10 years old), and one had six males kept in enclosures.

Use of concrete flooring in rest areas during the day was observed in 52% of camps, and for older camps, concrete paths often were used during trekking activities. A study of 1,422 elephants at tourist venues in Thailand by Schmidt-Burbach, Ronfot & Srisangiam (2015) also reported 25.6% were kept on concrete ground. It is known that elephants require varied substrates to provide for their physiological needs, and that standing for long periods of time on hard substrates can cause musculoskeletal disorders, including leg, foot, and nail problems (Buckley, 2008; Miller, Hogan & Meehan, 2016). Holdgate et al. (2016a) also found a relationship between flooring substrates and recumbent rest, an important welfare outcome. Elephants who spent any amount of time housed on all-soft substrate were recumbent 1.1 h more per day than those who were never on all-soft substrate. The AZA standards for elephant management and care (Association of Zoos and Aquariums (AZA), 2012) suggest that providing a combination of hard substrates to promote normal wear of footpads and soft substrates, such as earth and sand, to promote dust bathing is preferred. Floors should be able to be cleaned daily and quick to dry. It is possible that elephants are at an increased risk for foot and nail problems because of the amount of time they spend standing and walking on hard or rough substrates, and the lack of suitable foot care programs. Increased free foraging could reduce time spent on hard surfaces and reduce the time elephants are on chains.

### Nutrition

Determining the daily amount of food and water consumed by each elephant was beyond the scope of this study, but the survey gathered information on type of foods offered and estimated amounts of roughage. Elephants consume about 5% of their body weight (on wet matter basis), so an elephant cow would need as much as 150–175 kg, and a bull 200–275 kg of fodder per day (Natarajan, 1986). The average amount of roughage presented in this study (116 kg for adult males and 125 kg for adult females) was markedly lower than that, which could represent an under reporting because food was not weighed nor was intake recorded. However, all elephant camps provided supplements and 39% of camps permitted free-foraging periods. Corn stalks and grasses (napier and bana) were the main foodstuffs offered to elephants in these camps, whereas a previous study reported that napier grass, banana tree, and bamboo were used in this area (Godfrey & Kongmuang, 2009). In other range countries, elephants are fed other items, such as palm leaves, fresh grass (*Typha angustifolia, Alpinia nigra, Arundo donax, Tripsacum laxum, S. arundinaceum, S. narenga, Ochlandra* spp.), leaves of trees and shrubs in India (Joseph et al., 2013; Vanitha, Thiyagesan & Baskaran, 2008; Varma et al., 2010), leaves of *Ficus* spp. in Nepal (Varma & Ganguly, 2011), and meadow (*Spartina patens*) and alfalfa (*Medicago sativa*) hay in western zoos (Association of Zoos and Aquariums (AZA), 2012; British and Irish Association of Zoos and Aquariums (BIAZA), 2010). Choice of fodder often depends on local or seasonal availability. Corn stalk was fed in 85% of camps because northern Thailand is the largest maize-producing region, and corn stalks are an abundant byproduct (Ekasingh et al., 2004). Food variety or an ability to forage was limited in many elephant camps, however. Over half of the elephants (61% of camps) had no regular access to natural forage, even though they resided in camps located near a forest. Many local governments and communities have policies in place to protect forests, which includes restricting elephants. This raises questions about how many camps a region can effectively support, taking into consideration the need to provide adequate nutrition for elephants while protecting habitat.

In this study, drinking frequency of elephants was about three times per day, and 82% of camps had no water sources in rest areas during nighttime. In the wild, elephants generally do not have access to water at all times. African elephants are reported to drink once a day or only once every 3 or 4 days (Apps, 2000). Asian elephants are also reported to drink at least once a day and are never far from a permanent source of fresh water (Shoshani & Eisenberg, 1982). In western zoos, elephants are provided water ad libitum via continuously running sources or automatic watering devices throughout indoor and outdoor spaces (Association of Zoos and Aquariums (AZA), 2012; Clubb & Mason, 2002). There are no data on how often elephants need to drink, but because of the higher physical activity experienced by elephants in the tourist industry compared to wild and zoo elephants, it would be better to provide access to fresh water more often to maintain full health and vigor.

### Health care

A survey of captive elephant (mostly in tourist camps) health status in Thailand by the TECC identified a number of management-related problems, including injuries from use of controlling equipment, poor body condition and gastrointestinal issues (Angkawanish et al., 2009), so health care is critical for this population. However, up to 39% of surveyed elephants in northern Thailand did not have access to proper health care, especially those in small and medium camps that did not have an on-site veterinarian or access to regional vet services. Most (71%) large camps had at least one full-time veterinarian, and 12 camps had built clinics on-site. To aid small and medium camps, mobile clinics funded by the TECC, CMU, and DLD provide free health care, although responses can take hours or even days. One solution would be for several camps in an area to create a consortium to share the cost of a veterinarian and health care supplies. In May 2017, the Chiang Mai Elephant Alliance was formed, which is a group of elephant camp owners in the region that meets regularly to discuss elephant issues faced by the membership, including how camps can work together to provide better veterinary care (e.g., small camps working together to share a veterinarian, and supporting a mobile veterinary clinic). Additionally, more elephant hospitals are needed in Chiang Mai province to cover the distribution and density of elephant camps in this area. Training programs for mahouts on elephant health care have been conducted by the TECC and CMU (CMU, 2015), but not on a regular enough basis to cover every camp. Effective education and training programs for camp veterinarians, veterinary assistants, and mahouts on elephant medication and health care should be more developed and done regularly.

### Breeding activities

Many elephant camps (21%) in this study were not actively involved in breeding either because of a policy against it or the lack of breeding elephants and expertise. According to Thitaram (2012), due to their endangered status, captive breeding of Asian elephants in range countries should be encouraged, and is important to sustain captive populations, improve education, strengthen human–elephant relations, and potentially to provide animals for reintroduction back into the wild. In 1997, a reintroduction project was initiated under the HRH Queen Sirikit of Thailand, with Angkavanish & Thitaram (2012) reporting that former tourist elephants can survive in the forest and successfully reproduce.

Breeding success of captive elephants in Asia is variable and many populations are not self-sustaining (Thitaram, 2012). Major factors responsible for poor reproduction in Thailand are lack of breeding interest by bulls or cows, and poor estrous detection. Because of the higher numbers of females, especially in the smaller camps, lack of breeding bulls is an issue. Population sustainability could be compromised if non-breeding camps have high numbers of reproductive age females that do not contribute to the gene pool. In addition, lack of breeding and long periods of reproductive inactivity can reduce fertility even in young females due to the development of reproductive tract pathologies (Hermes, Hildebrandt & Goritz, 2004). However, it is equally important that captive breeding only be conducted by facilities with appropriate space and expertise to ensure good management of breeding elephants and resulting offspring. Older camps have more males, probably because the mahouts possess the knowledge and experience to manage bulls, which leads to better breeding success. Bulls, especially in musth, are difficult to handle, and caring for them is a specialty within the mahout profession (Vortkanp, 2006). Sukumar (2003) stated that even when male and female elephants are kept together, such as in logging or forest camps, the reproductive ability of a breeding female may be compromised by a heavy workload and poor nutrition. Moreover, a study of logging elephants found that females born in high stress months (i.e., high workload for working elephants and monsoon conditions associated with increased glucocorticoid concentrations) exhibited earlier reproductive senescence and significantly reduced lifetime reproductive success than counterparts born at other times of year (Mumby et al., 2015). Therefore, elephant camps might increase breeding success by providing breeding elephants the space and time to allow them to be together, minimizing workloads, and maintaining good nutrition and body condition. It is important that elephant owners, mahouts, and veterinarians all be involved in breeding plans to ensure cooperation and proper management.

### Mahout management

Mahouts share a relationship with elephants that is rarely matched in other human–animal interactions. As a result, they play a very important role in the welfare and health status of elephants (Cooper & Cooper, 2013; Hart, 1994). A survey of 42 privately owned safari elephants in Nepal found there was a direct relationship between the welfare of elephants and that of their mahouts (Vries, 2014), while Hart & Sundar (2000) found that a program of regular structured work for elephants and their mahouts was important for the welfare of both. Ward & Melfi (2015) demonstrated that keeper-animal dyads were formed in zoos, and keepers have different stockmanship styles, which are influenced by attitude, knowledge of the animals in their care, and work experience. In the same way, mahouts who have a good attitude about their job, greater knowledge and experience, and more appreciation of elephants likely take better care of them, which would lead to better welfare.

The mahout’s welfare is an indication of elephant welfare, and mental health and physical fitness of mahouts are primary requirements to control an elephant (Radhakrishnan, Rajeev & Radhakrishnan, 2011; Vanitha, Thiyagesan & Baskaran, 2009). It is clear that many of the mahouts in this study did not get a suitable salary, insurance, or benefits. The mean monthly salary was about 8,000 baht (<$250), up from 3,000–5,000 baht a decade ago (Kontogeorgopoulos, 2009a). Mahouts receive tips directly from tourists in 67% of camps, but that source of income is unpredictable. Based on a minimum rate set by the Ministry of Labour in Thailand, the current employee salary should be 300 baht a day, which equates to 9,000 baht a month (Thangstapornpong & Porananond, 2017). Mahout salaries should be even higher, because they often work 7 days a week. There is also a need for better health care and accident insurance for mahouts because it a risky profession, particularly for those who work with aggressive elephants. Mahout welfare has been intensively investigated in India, where underpayment, financial instability, and insufficient insurance coverage are continuing problems (Radhakrishnan, Rajeev & Radhakrishnan, 2011; Vanitha, Thiyagesan & Baskaran, 2009; Varma, 2015). A survey of 45 veterinarians in eight Asian elephant range countries revealed that the health of mahouts was routinely screened in only 29% of the respondents. Most respondents indicated a need for improved mahout training in the areas of daily routine health, elephant biology and behavior, elephant restraint and handling tools, and drug use (Miller et al., 2015). Elephant camps should provide a good living and quality of life for mahouts to make the career more attractive.

## Conclusions

Elephant camps are now an important part of the tourism industry in Thailand, and provide the only viable and realistic options for sustaining a captive population and providing employment for mahouts. However, elephant management and care varies considerably. Activities provided by elephant camps appear to be changing from more intensive activities, like riding with saddles and shows, to more intimate experiences, like feeding, bathing and walking. Because there are no enforced guidelines or standards for elephant camps to follow, each facility manages elephants to meet their own criteria or financial limitations, and as a result not all meet elephant welfare needs. The results of this study will be used to guide future studies on elephant welfare in Thailand and other elephant venues. The next step is to create variables for subsequent multivariable analyses to determine what management factors are related to health and welfare outcomes of elephants, and develop science-based guidelines or standards to aid management of elephants used in tourism. It should be noted that we surveyed only about 18% of the captive elephant population in Thailand, so expanding this study to other populations across the country is needed to more fully understand the variability in elephant management practices. In addition, understanding of the situation in Thailand is valuable for other Asian countries. Myanmar, for example, is reducing logging country-wide, which means that elephants used in the logging industry will soon be out of work (Jaysinghe & Soe, 2017), and so that country will face the same problems that Thailand did just a few decades ago.

## Supplemental Information

10.7717/peerj.5996/supp-1 Supplemental Information 1 Questionnaire sheet used to record information during camp visits.

10.7717/peerj.5996/supp-2 Supplemental Information 2 Raw data. Raw data for Tables 1–7.

10.7717/peerj.5996/supp-3 Supplemental Information 3 Table S1. Number and percentage (in parentheses) of elephant camps for each years of camp operation and size of camp by roughage purchase. *Chi-square tests of association.

10.7717/peerj.5996/supp-4 Supplemental Information 4 Table S2. Number and percentage (in parentheses) of elephant camps for each years of camp operation by free foraging opportunities. *Significant at *P* < 0.05 between two variables using Chi-square tests of association.

10.7717/peerj.5996/supp-5 Supplemental Information 5 Table S3. Number and percentage (in parentheses) of elephant camps for each years of camp operation and size of camp by having veterinarians. *Significant at *P* < 0.05 between two variables using Chi-square tests of association.

10.7717/peerj.5996/supp-6 Supplemental Information 6 Table S4. Number and percentage (in parentheses) of elephant camps for each years of camp operation and size of camp by having a clinic.

10.7717/peerj.5996/supp-7 Supplemental Information 7 Table S5. Number and percentage (in parentheses) of elephant camps for each years of camp operation and size of camp by breeding success. *Significant at *P* < 0.05 between two variables using Chi-square tests of association.

10.7717/peerj.5996/supp-8 Supplemental Information 8 Table S6. Mean (± SE) mahout salary of elephant camps based on years of camp operation, size of camp, and type of work.

## Additional Information and Declarations

### Data Availability

The following information was supplied regarding data availability: The raw measurements are provided in the Supplemental Files.
