# Supplementary material for: Changing trends in elephant camp management in northern Thailand and implications for welfare
Source: PeerJ. 2018 Nov 23;6:e5996. doi: 10.7717/peerj.5996 (PMC6254247; doi:10.7717/peerj.5996)
Supplement: Supplemental Information 1 [file peerj-06-5996-s001.pdf]

Supplement 1

Questionnaire sheet used to record information during camp visits

Name of interviewer ....

Name of interviewee ……………………………………… position.......................................

Date……………..............

Section 1. Camp and elephant information

Camp name …………………………………………………………………

Address ……………………………………………………………………..

Years of operation ....

Location:

close to village	□ no	□ yes	
close to main road	□ no	□ yes	
close to river/stream	□ no	□ yes	
close to forest	□ no	□ yes:	type of forest ……………………
close to agricultural area	□ no	□ yes:	specify ....
close to industry area	□ no	□ yes:	specify ....

Activity with elephants

□ riding with a saddle □ riding bareback □ no riding □ elephant show

□ free movement □ feeding □ bathing □ others ……………………………………...

Other activities

□ ox-cart riding □ bamboo rafting □ rubber rafting □ visiting hill tribe villages

□ trekking □ zip lines □ restaurant □ gift shop □ others …………………...

Program for tourists

□ half day □ full day: How many days? ………………… days

□ overnight □ others ……………………………………..

Other animals in the camp

□ no □ yes: □ ox □ buffalo □ dog □ cat □ chicken □ others ……………………

Elephant number ………………… elephants

□ male: How many? ……………………… elephants

□ female: How many? ……………………… elephants

Age of elephants

Age (years)	Number of elephants	
	Male	Female
□ 0 - 3		
□ 4 - 10		
□ 11 - 20		
□ 21 - 30		
□ 31 - 40		
□ 41 - 55		
□ > 55		

Owner of elephants

□ camp: How many elephants? ……………………… elephants

□ mahout: How many elephants? ……………………… elephants

□ others: How many elephants? ……………………… elephants

Section 2. Elephant management

Working

Operation time	normal	start time …………….
		end time …………….
	high tourist season	start time …………….
		end time …………….
	low tourist season	start time …………….
		end time …………….

Walking

Distance and duration of walking

Type of work/activity	Distance/round (meters)	Duration/round (minutes)	Round/day (round)		
			Normal	High tourist season	Low tourist season
□ riding with a saddle					
□ riding bareback					
□ no riding					
□ elephant show					
□ free movement					
□ others: specify …………………...……					

Walking trail

Type of work/activity	Type of trail					
	Floor (ground/ concrete/etc.	Flat (yes/no)	Slope (yes/no)	River (yes/no)	Road (yes/no)	Others ……..
□ riding with a saddle						
□ riding bareback						
□ no riding						
□ elephant show						
□ free movement						
□ others: specify ……………….…......						

(If you have a riding bareback program)

How many elephants in this program? …….. elephants: male …..….. female ………

How many tourists per elephant? ………………. persons

Where can tourists sit on the elephant? □ neck □ back

Where are mahouts? □ sit on a neck □ sit on a back □ walk with elephants

Are elephants attached by a cinch?

□ no □ yes: What type? □ rope □ rubber tube □ others ……………………

How long of this program? ……………….. minutes/hours

(If you have a riding with a saddle program)

How many elephants in this program? …….. elephants: male ……… female ………

How many tourists per elephant? ………………. persons

What are saddles made from?

□ wood: How many weight? …………… kg

□ metal: How many weight? …………… kg

□ rattan: How many weight? …………… kg

□ others ………….. How many weight? …………… kg

What types of bedding? □ gunnysack □ hammered bark of tree □ blanket

□ sponge □ plastic □ others ………………………..

How many weight of bedding? …………… kg

What equipment are used to tie saddles? □ rope □ rubber tube □ others ………….

Where do you tie equipment to elephants? □ neck □ chest □ tail

Are saddles removed during break time? □ no □ yes: How long? ………………......

How long of this program? ……………….. minutes/hours

(If you have a no riding program)

How many elephants in this program? …….. elephants: male ……… female ………

How many tourists per elephant? ………………. persons

How long of this program? ……………….. minutes/hours

(If you have an elephant show)

How many elephants in this program? …….. elephants: male ……… female ………

How many tourists per round? ………………. persons

How long of a show? ……………….. minutes

What kind of activities in a show?

□ open mouth □ playing musical instruments

□ sit □ kicking soccer balls

□ lie down □ dancing to music

□ lift up a leg □ painting

□ raise a trunk □ demonstrating logging skill

□ others …………………………………………………………………………

(If you have a free movement program)

How many elephants in this program? …….. elephants: male …….. female ………

How many tourists per round? ………………. persons

How far of tourists from elephants? ……………… meters

Can tourists touch elephants? □ no □ yes

How can mahouts control their elephant? ……………………………………………...

How long of this program? ……………….. minutes

Break time

Type of work/activity	Break time	
	When? (at noon, between rounds, etc.)	How long? (minutes)
□ riding with a saddle		
□ riding bareback		
□ no riding		
□ elephant show		
□ free movement		
□ others: specify ………………….....……		

Equipment used during working

Do you have any equipment to control elephants during working? □ no □ yes:

Type of equipment	How many elephants?	
	Every elephants	Some elephants: specify …………….…..
□ hook		
□ nail		
□ knife		
□ slingshot		
□ chain		
□ others: specify ……………………		

Do elephants have a day off? □ no □ yes: specify ……………………………………………….

Is there any injury/problem caused from working?

□ no □ yes: cause □ restraint equipment □ elephant fight

□ saddle equipment □ weakness from workload

□ walking trail □ others …………………………

Rest area

Rest area during daytime

Type of rest area	Movement (chain/no chain)	Floor type (ground/concrete/others)	How many elephants?
□ conventional housing			
□ enclosure			
□ under tree			
□ open space			
□ grass field			
□ others: specify ……………………			

Rest area during nighttime

Type of rest area	Movement (chain/on chain)	Floor type (ground/concrete/others)	How many elephants?
□ conventional housing			
□ enclosure			
□ under tree			
□ open space			
□ grass field			
□ others: specify ……………………			

(If you have housing)

What are housing made of? □ wood □ concrete □ metal □ others ………………

Height …………………. meters

Roof type: □ zinc □ tile □ grass □ others ………………………..

Can water drain out easily? □ no □ yes

Is there any rough surface? □ no □ yes

(If you have the enclosure)

How large of enclosures? …………………… square meters

How many elephants in each enclosure? ………………….. elephants

How do you clean rest areas?

□ remove dung: How often? ………………………..

□ wash: How often? ………………………..

□ scrub: How often? ………………………..

Chaining

(If you chain elephants)

Length of chain during daytime …………………… meters

Length of chain during nighttime …………………… meters

Size of chain ………………… unit

What kind of materials you chain elephants to? □ pin □ pole □ tree □ others ………………

Nutrition

Type, source, freshness and storage of food

Type	Source	Freshness	Storage
□ grass: specify type			
□ corn stalk			
□ pineapple stalk			
□ bamboo			
□ hey			
□ banana			
□ sugar cane			
□ sticky rice			
□ tamarind			
□ pellet feed			
□ others: specify ……………………<br>……………………<br>……………………<br>……………………<br>……………………			

Feeding

Elephant category	Type of food	Amount/day	Times/ day	Amount/time
Pre-wean				
Young<br>(wean – 10 years)				
Adult males				
Adult females				
Old<br>(> 55 years)				
Musth				
Pregnant				

Free foraging

□ no: Why? …………………………………………………………………......

□ yes: Where? □ forest □ grass field □ others ………………………..

When? □ daytime □ nighttime □ others ……………………..

How often? □ every day □ others ………………………..

How many elephant? ……………………… elephants

Water

Water for bathing

Source of water □ river/stream □ underground water

□ tap water □ others ………………………..

Water processing □ no process □ filtration □ others ………………...

Water for drinking

Source of water □ river/stream □ underground water

□ tap water □ others ………………………..

Water processing □ no process □ filtration □ others ……………..…...

Where do elephants drink water? □ river/stream □ pond □ bucket

□ tap □ others ………………………..

Do you clean water containers? □ no □ yes: How often? ……………………...

How can elephant drink water during daytime? ………..………………………

How can elephant drink water during nighttime? ……………………………...

How many times elephant can drink water? …………… times/day

Health care

Bathing

How often do mahouts bath their elephants? .... times/day

When do mahouts bath their elephants? ……………………………….

Healthcare staff

□ no □ yes:	□ veterinarian	How many? ……………
	□ animal husbandman	How many? ……………
	□ vet nurse	How many? ……………
	□ others …………………..	How many? ……………

Elephant clinic

□ no □ yes

Medicine and equipment

□ wound dressing	□ supplement
□ eye drops	□ systemic antibiotic
□ local anti-inflammation	□ systemic anti-inflammation
□ foot trimming	□ others …………………………………………………

What do you do if you have sick elephants?

□ treat by camp staffs

□ consult : Who? ………………………………………….

□ contact mobile clinic: Which one? ……………………………………….

□ send elephants to a hospital: Where? …………………………………….

Transportation: □ camp's truck □ hired truck

Health supplement

□ no □ yes: □ commercial herbs □ herbs from forest □ vitamin □ others ………

Annual health check program

□ no □ yes: □ by camp: How often? ………………… times/year

What program? …………………………………………

□ by government staffs: How often? ………………… times/year

What program? …………………………

Deworming program

□ no □ yes: How many times? ………………… times/year

Vaccination program

□ no □ yes: What kind of vaccine? ………………………..

How many times? ………………… times/year

Foot care

□ no □ yes: How? ………………………………………………………………...

How often? …………………………

Quarantine

□ no □ yes: How? ………………………………………………………………...

How long? ……………………….. days

Program: □ deworming □ vaccination □ blood test □ others ………...

Breeding

Breeding elephant

□ no □ yes: bull …………… elephants

dam …………... elephants

Breeding program

□ no: Why? ………………………………….……………………………………..

□ yes

(If you have breeding program)

At what age do you start to breed elephants? ………………… years old

Heat detection

□ no □ yes: How? □ by mahouts

□ by breeding bulls: □ direct detection □ from urine samples

□ by hormone level

What do you do if females are considered to be in estrus?

□ breed in the camp during free time/after work

□ stop working and separate to breed: Where? …………………………

How long? ……………….. days

□ others …………………………………………….

Do breeding elephants have a chance to be together?

□ no □ yes: How? ……………………………………………………………………

Do you bring your elephants to breed at other camps?

□ no □ yes: Where? …………………………….……………….……

Do you bring elephants from other camps to breed at your camp?

□ no □ yes: from where? ………………………………….……

Do breeding elephants work the same as other elephants?

□ yes □ no: □ not work □ work less than other elephants: specify …………………...

(If you have adult females at the age of 15-25 years old)

Have they ever breed?

□ no □ yes: How many? ……………….. elephants

□ not found pregnant

□ considered pregnant: How may? ……………….. elephants

How many calves that born from camp breeding program? ………….. elephants

How many calves that born from camp breeding program in the last 5 years? ….. elephants

Breeding record:

□ no □ yes: □ date □ name of breeding elephants

□ number of breeding □ others ………………..

Pregnant and baby elephant

Have you had pregnant elephants and/or calves? □ no □ yes

(If you have had pregnant elephants and/or calves)

How do you know that elephants are pregnant?

□ physical changes: □ breast □ abdomen □ others …………………………………...…

□ behavioral changes: specify ………………………………………………………….

□ hormone level: by who? ………………………….

□ ultrasound: by who? …………………………

Is there any staff who have experience about pregnant elephants and calves?

□ no □ yes: How many? ……………….. staffs

Pedigree

□ no □ yes

How do pregnant elephants work?

□ work normally until parturition

□ stop working: At what stage? …………………………….……

□ reduce workload: How? ……………………………………………
At what stage? …………………………….……

Specific zone for parturition

□ no □ yes: Where? …………………………………….

Helper mother during parturition

□ no □ yes

Staff during parturition

□ no □ yes

Problem about pregnant elephants and calves in the last 5 years

□ no □ yes: □ dystocia: How many cases? ……………… cases
predisposing cause ……………………………………...

□ stillbirth: How many cases? ……………… cases
predisposing cause ……………………………………...

□ calve rejection: How many cases? ……………… cases
predisposing cause ……………………………...

□ others ………………………………………………………………

Nursery zone

□ no □ yes: Where? …………………………………
Is there a fence? □ no □ yes

Weaning

□ no □ yes: How old? …………. years old

Do you separate calves from mothers?

□ no □ yes: How old? …………. years old
Procedure …………………………………………………………

Training

□ no □ yes: How old? …………. years old

How long? …………… months

Who is a trainer? ………………………..

What are training tasks? ……………………………………………..

Have you found any problem from training? …………….…………….

Health problem in calves

□ no □ yes: □ diarrhea □ bloat □ constipation □ skin problem □ wound

□ eye problem □ EEHV □ others ………….…………………………

Old elephant

Have you had old elephants? □ no □ yes

(If you have had old elephants)

Do old elephants work the same as other elephants?

□ yes □ no: □ not work

□ work less than other elephants: specify …………………..................

□ work in different program: specify …………………………………..

□ others ……………………………………….

Health problems

□ no □ yes: □ bloat □ constipation □ skin problems □ wound

□ foot problem □ stiffness □ arthritis □ abnormal gait

□ cataract □ blindness □ tooth lost □ others ....

Retired elephant

Have you had retired elephants? □ no □ yes

(If you have had retired elephants)

How many retired elephants? ……………… elephants

Where do they live? …………………………………………..

Do they have chance to exercise? □ no □ yes: How? ……………………………………………

Musth elephant

Have you had musth elephants? □ no □ yes

(If you have had musth elephants)

What do you do when elephants are considered to be in musth condition?

□ breed

□ stop working

□ separate to a specific zone: Where? ……………………………..

How do they eat food? …………………………………

How do they drink water? ……………………………...

chain: □ no □ yes: How long? ………………. meters

other equipment: □ no □ yes: specify …………………

How long? ……………………………………………...

□ others ……………………………………………………………………………

Is there any plan for emergency cases?

□ no □ yes: □ sedative

□ dart gun and equipment

□ experienced staffs

□ others …………………………

Musth record

□ no □ yes: □ date □ duration □ signs □ others ……………………………………

Section 3. Mahout management

Number of mahouts …………….. mahouts

□ permanent (regular) mahout …………….. mahouts

□ reserve mahout ……………. mahouts

Number of mahout per elephant …………………

Do you have enough mahouts?

□ yes □ no: Why? ……………………………………………………………….

Ethnic of mahouts

□ native of Thailand

□ ethnic groups of Thailand: specify ……………………………..

□ native of Myanmar

□ ethnic groups of Myanmar: specify ……………………………

□ others ………………………………………..

Benefit

Salary ……………………. baht/month

Other income: □ tips: How much? ……………………….

□ round of working: How much? ………………………..

□ others ……………………………………………………………………

Insurance □ no □ yes: specify …………………………………………………….

Health check □ no □ yes: specify …………………………………………………….

Others ………………………………………………………………………………..

Day off

□ no □ yes: How many?.............................. days/month

What are important criteria for mahout employment?

□ used to be a mahout

□ recommended from reliable persons

□ can communicate in Thai language

□ have a legal document (for ethnic groups)

□ others ……………………………………………………………………………

Have you sent mahouts to join training courses?

□ no: Why? …………………………….....……………………………………..

□ yes: What courses? …………………………………………………………….

How often? …………………………

Rule ……………………...…..……………………………………...……………………..

Punishment ……………………………………………………………………………..
