# Supplementary material for: Changing trends in elephant camp management in northern Thailand and implications for welfare
Source: PeerJ. 2018 Nov 23;6:e5996. doi: 10.7717/peerj.5996 (PMC6254247; doi:10.7717/peerj.5996)
Supplement: Supplemental Information 3 — *Chi-square tests of association. [file peerj-06-5996-s003.docx]

**Table S1.** Number and percentage (in parentheses) of elephant camps for each years of camp operation and size of camp by roughage purchase.

|  |  |  | Roughage Purchase | |  |
| --- | --- | --- | --- | --- | --- |
| Variable |  | Camp N | Yes | No | P* |
| Years of Operation | 0-5 | 10 | 1 (25%) | 9 (31%) | 0.056 |
|  | 6-15 | 14 | 0 (0%) | 14 (48%) |  |
|  | >16 | 9 | 3 (75%) | 6 (21%) |  |
| Size of Camp | Small | 16 | 13 (45%) | 3 (75%) | 0.434 |
|  | Medium | 10 | 9 (31%) | 1 (25%) |  |
|  | Large | 7 | 7 (24%) | 0 (0%) |  |

*Chi-square tests of association.
