# Supplementary material for: Changing trends in elephant camp management in northern Thailand and implications for welfare
Source: PeerJ. 2018 Nov 23;6:e5996. doi: 10.7717/peerj.5996 (PMC6254247; doi:10.7717/peerj.5996)
Supplement: Supplemental Information 4 — *Significant at P < 0.05 between two variables using Chi-square tests of association. [file peerj-06-5996-s004.docx]

**Table S2.** Number and percentage (in parentheses) of elephant camps for each years of camp operation by free foraging opportunities.

|  |  |  | Free Foraging | |  |
| --- | --- | --- | --- | --- | --- |
| Variable |  | Camp N | Yes | No | P |
| Years of Operation | 0-5 | 10 | 8 (62%) | 2 (10%) | <0.007* |
|  | 6-15 | 14 | 3 (23%) | 11 (55%) |  |
|  | >16 | 9 | 2 (15%) | 7 (35%) |  |

*Significant at P<0.05 between two variables using Chi-square tests of association.
