# Supplementary material for: Changing trends in elephant camp management in northern Thailand and implications for welfare
Source: PeerJ. 2018 Nov 23;6:e5996. doi: 10.7717/peerj.5996 (PMC6254247; doi:10.7717/peerj.5996)
Supplement: Supplemental Information 6 [file peerj-06-5996-s006.docx]

**Table S4.** Number and percentage (in parentheses) of elephant camps for each years of camp operation and size of camp by having a clinic.

|  |  |  | Having a Clinic | |  |
| --- | --- | --- | --- | --- | --- |
| Variable |  | Camp N | Yes | No | P* |
| Years of Operation | 0-5 | 10 | 1 (8%) | 9 (43%) | 0.096 |
|  | 6-15 | 14 | 6 (50%) | 8 (38%) |  |
|  | >16 | 9 | 5 (42%) | 4 (19%) |  |
| Size of Camp | Small | 16 | 2 (17%) | 14 (67%) | <0.001* |
|  | Medium | 10 | 3 (25%) | 7 (33%) |  |
|  | Large | 7 | 7 (58%) | 0 (0%) |  |

*Significant at P < 0.05 between two variables using Chi-square tests of association.
