# Supplementary material for: Changing trends in elephant camp management in northern Thailand and implications for welfare
Source: PeerJ. 2018 Nov 23;6:e5996. doi: 10.7717/peerj.5996 (PMC6254247; doi:10.7717/peerj.5996)
Supplement: Supplemental Information 7 — *Significant at P < 0.05 between two variables using Chi-square tests of association. [file peerj-06-5996-s007.docx]

**Table S5.** Number and percentage (in parentheses) of elephant camps for each years of camp operation and size of camp by breeding success.

|  |  |  | Breeding Success | |  |
| --- | --- | --- | --- | --- | --- |
| Variable |  | Camp N | Yes | No | P* |
| Years of Operation | 0-5 | 10 | 2 (11%) | 8 (54%) | 0.026* |
|  | 6-15 | 14 | 9 (50%) | 5 (33%) |  |
|  | >16 | 9 | 7 (39%) | 2 (13%) |  |
| Size of Camp | Small | 16 | 5 (28%) | 11 (73%) | 0.009* |
|  | Medium | 10 | 6 (33%) | 4 (27%) |  |
|  | Large | 7 | 7 (39%) | 0 (0%) |  |

*Significant at P < 0.05 between two variables using Chi-square tests of association.
