# Supplementary material for: Changing trends in elephant camp management in northern Thailand and implications for welfare
Source: PeerJ. 2018 Nov 23;6:e5996. doi: 10.7717/peerj.5996 (PMC6254247; doi:10.7717/peerj.5996)
Supplement: Supplemental Information 8 [file peerj-06-5996-s008.docx]

**Table S6.** Mean (± SE) mahout salary of elephant camps based on years of camp operation, size of camp, and type of work.

| Variable |  | Camp N | Mean Mahout Salary (baht) |
| --- | --- | --- | --- |
| Years of Operation | 0-5 | 10 | 7,143 ± 584.7 |
|  | 6-15 | 14 | 8,727 ± 501.7 |
|  | >16 | 9 | 7,250 ± 612.4 |
| Size of Camp | Small | 16 | 7,192 ± 342.2 |
|  | Medium | 10 | 8,714 ± 653.4 |
|  | Large | 7 | 8,250 ± 997.9 |
| Type of Work | Riding with a Saddle | 7 | 7,875 ± 965.6 |
|  | Riding with a Saddle and Show | 5 | 6,333 ± 881.9 |
|  | Riding Bareback | 10 | 8,722 ± 565.9 |
|  | Riding with a Saddle and Riding Bareback | 5 | 7,300 ± 768.1 |
|  | No Riding | 5 | 7,375 ± 898.5 |
|  | Observation | 1 | 9,000 |
